# Viral RNA and infectious virus in mucosal specimens from guinea pigs modelling early phases of lethal and non-lethal Lassa fever

**DOI:** 10.1080/22221751.2022.2071637

**Published:** 2022-05-23

**Authors:** Stephen R. Welch, Sarah C. Genzer, JoAnn D. Coleman-McCray, Jessica R. Harmon, Florine E.M. Scholte, Joel M. Montgomery, Christina F. Spiropoulou, Jessica R. Spengler

**Affiliations:** Viral Special Pathogens Branch, Division of High Consequence Pathogens and Pathology, Centers for Disease Control and Prevention, Atlanta, GA, USA

**Keywords:** Lassa virus, arenavirus, viral hemorrhagic fever, virus isolation, body fluids, mucosal swab, shedding, transmission

## Abstract

Lassa fever (LF) is endemic to broad regions of West Africa. Infection with Lassa virus (LASV), the etiologic agent of LF, results in a spectrum of clinical signs in humans, including severe and lethal hemorrhagic disease. Person-to-person transmission occurs through direct contact with body fluids or contaminated bedding and clothing. To investigate transmission risk in acute LASV infection, we evaluated viral RNA and infectious virus obtained from conjunctival, nasal, oral, genital, and rectal swab specimens from guinea pigs modelling lethal and non-lethal LF. Viral RNA and infectious virus were detected in all specimen types beginning 8 days post infection, prior to onset of fever. In the pre-clinical and clinical period, virus was isolated from a subset of nasal, oral, genital, and rectal swabs, and from all conjunctival swabs. Overall, conjunctival and nasal specimens most frequently yielded infectious virus. These findings indicate mucosal transmission risk based on virus isolation from various sites early in infection and support potential utility of minimally invasive specimen evaluation by RT-qPCR for LASV diagnostics.

Lassa virus (LASV), the etiologic agent of Lassa fever (LF), causes over 100,000 human cases annually in endemic regions of West Africa [1]. To date, mucosal shedding kinetics have not been extensively characterized in human cases or disease models, particularly during early infection. Lethal and non-lethal LF can be modelled using non-rodent-adapted clinical isolates of LASV in inbred strain 13/N guinea pigs [2,3]. Using this model, here we evaluate the presence of viral RNA and infectious virus in 5 mucosal sites at 5 timepoints early in the course of infection, representing the pre-clinical period and the period during which non-specific signs may be present, when transmission risk in humans would be highest as the index of suspicion is low. Additionally, to determine if shedding varies with disease outcome, we used two LASV strains: one that causes lethal disease in guinea pigs (Josiah) and one that causes non-lethal clinical disease (Sauerwald).

Groups of 25 strain 13/N guinea pigs (males and females; aged 365–1021 days) housed in a biosafety level 4 (BSL-4) laboratory were infected subcutaneously (SC) in the interscapular region with target dose 1 × 10^4^ FFU of LASV strain Josiah or Sauerwald (actual dose: 1.3 × 10^4^ FFU Sauerwald; 1.9 × 10^4^ FFU Josiah). Sauerwald was isolated from a fatal human case (clade II; GenBank: MG812680.1, MG812681.1) and grown on Vero-E6 cells (MOI 0.01, harvested 5–6 days post infection [pi]) [3]. Generation of recombinant Josiah, based on the sequence from a fatal human case, has been described previously [4] (clade IV; GenBank: HQ688673.1, HQ688675.1). Viral titres were calculated as focus-forming units (FFU) in Vero-E6 cells. All viral stocks were verified by sequencing and confirmed as mycoplasma-free by MycoAlert Plus reagents (Lonza).

Guinea pigs that develop clinical disease after LASV infection demonstrate early non-specific signs (e.g. injected sclera, anorexia, weight loss) and elevated temperatures peaking ∼12 dpi [3]. Fever resolves in both lethal and non-lethal cases, but animals with lethal disease exhibit progressively severe weight loss and clinical signs, including respiratory insufficiency, weakness/ataxia, and marked hypothermia, necessitating euthanasia ∼17–26 dpi. To investigate viral shedding early in infection, groups of 5 animals (3 females, 2 males) infected with each LASV strain were humanely euthanized at 1 of 5 pre-determined endpoints representing: a pre-clinical period (2 and 4 dpi); onset of mild, non-specific signs (8 dpi); peak temperature elevation (12 dpi); and progression to lethal disease or resolution of non-lethal disease (16 dpi).

All animals were monitored daily. At the predetermined timepoints, weights, temperatures and clinical score were recorded (Table S1 and S2). Terminal intracardiac blood was collected in EDTA from animals under deep isoflurane anesthesia followed by euthanasia (intracardiac sodium pentobarbital solution). Mucosal specimens were collected immediately post mortem from conjunctiva, nares, oropharynx, genitalia (intravaginal or preputial), and rectum. Sterile 6″ mini-tip (0.078″ diameter) polyester swab applicators (Puritan; cat. no. 25-800 1PD 50) were used for nasal specimens; sterile 6″ polyester-tipped applicators (Fisher Scientific; ref. 22363164) were used for all other sites. Two specimens were collected per site: one in 500 µL MagMAX lysis solution (1:1 with isopropanol) for RNA isolation, and one in 1 mL sterile DMEM (2× penicillin/streptomycin and 2× antifungal/antimycotic [both Gibco]) for virus isolation and quantification.

RNA from collected tissues (blood, eye, lung, gonad) and swabs was isolated using MagMAX Pathogen RNA/DNA Kit (RNA eluted in 75 µL; Thermo-Fisher Scientific). Genomic DNA was removed using BaseLine Zero DNase (Epicentre), and RT-qPCR was performed using SuperScript III Platinum One-Step RT-qPCR Kit (Invitrogen) with strain-specific primers and probe targeting LASV N gene. Standard curves generated by S-segment in vitro transcripts were used to quantify viral RNA (Figure 1(A); Table S1; Table S2). No viral RNA was detected 2 and 4 dpi in swabs or associated tissues. Viremia was first detected 4 dpi at low levels in both Josiah (3 of 5) and Sauerwald (2 of 5) infected animals. Beginning 8 dpi, a subset of all swab specimen types was positive in animals from both experimental groups, and viral RNA was detected in at least two swabbed sites in each animal. Viral RNA was detected in 73 of 75 (97%) swabs collected ≥8 days from Josiah-infected animals (23 of 25 [92%] 8 dpi and 25 of 25 [100%] 12 and 16 dpi). The two negative samples were genital swabs from males collected 8 dpi. Fewer swabs from Sauerwald-infected animals were positive: 55 of 75 (73%) swabs collected ≥8 dpi (18 of 25 [72%] 8 dpi; 20 of 25 [80%] 12 dpi; and 17 of 25 [68%] 16 dpi). Genital specimens were the only type that differed significantly by sex (female > male; *p* = 0.0036). In general, RNA levels were highest in genital followed by conjunctival and nasal specimens (oral and rectal were lowest). Relative RNA values and positivity coincided with peak viremia and body temperature elevation (12 dpi); levels remained stable 16 dpi with Josiah infection but declined with Sauerwald.

**Figure 1.**
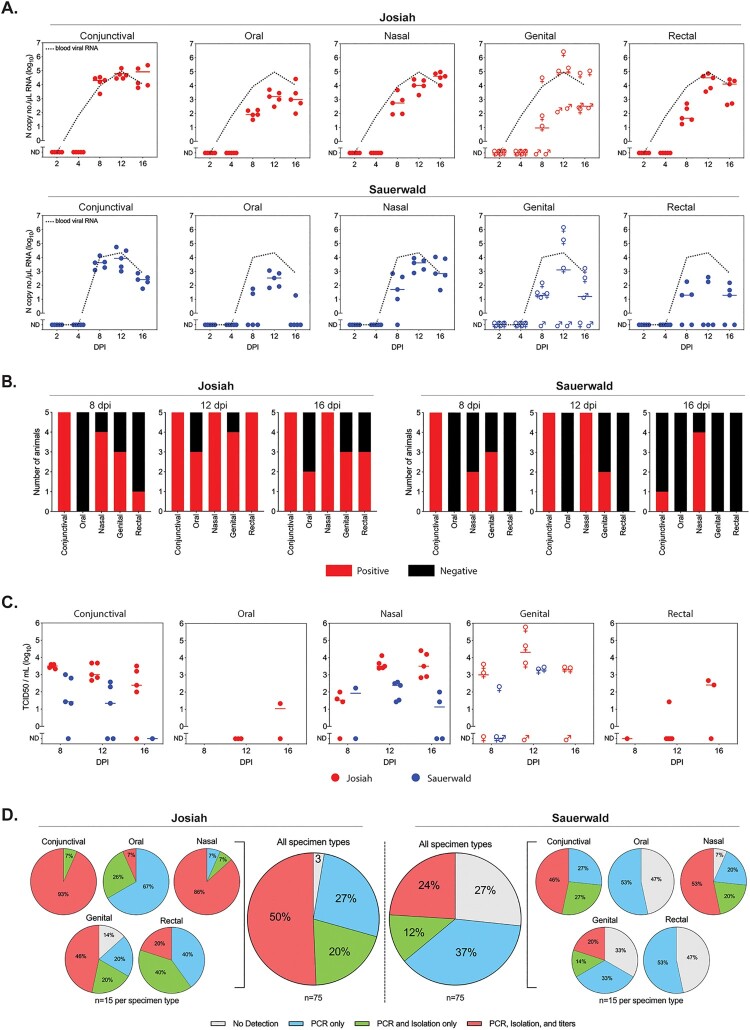
Analysis of mucosal specimens from Lassa virus-infected strain 13/N guinea pigs. Groups of 25 animals were inoculated subcutaneously with LASV strain Josiah or strain Sauerwald (target dose: 1 × 10^4^ FFU), and 5 animals per experimental group were serially euthanized at 1 of 5 pre-determined timepoints. Five paired swab specimens were collected per animal, one for RT-qPCR and the other for virus isolation and titration. (A) Levels of LASV RNA (N gene copy no./µL) in individual swab specimens are represented as dots (line at median), and median levels in EDTA whole blood specimens from groups of animals serially euthanized at the indicated timepoints are shown as dotted black line. For genital samples, values from samples collected from females (♀; intravaginal) and males (♂; preputial) are indicated. (B) Virus isolation was attempted with all swab specimens collected 8, 12, and 16 days post infection (dpi; *n* = 150). Red indicates proportion of samples from which virus was isolated; black indicates proportion of samples without isolatable virus. (C) Virus titration (TCID_50_/mL) was performed on all swab specimens collected 8, 12, and 16 dpi from which virus was isolated (*n* = 80). Some isolate-positive samples were below limit of detection in titration assays. For genital samples, values from females (♀; intravaginal) and males (♂; preputial) are indicated. (D) Summary of sample analyses. Total proportion of swab samples obtained 8, 12, and 16 dpi from Josiah- (*n* = 75) or Sauerwald-infected (*n* = 75) animals with no LASV detected (grey), or LASV detected by PCR alone (blue), PCR and virus isolation (green), or PCR, virus isolation, and virus titration (red). Samples are further broken down by both strain and specimen type, representing the 15 individual specimens for each sample type analysed. ND, not detected.

Presence of infectious virus was determined for all swab specimens collected 8, 12, and 16 dpi; 100 µL of specimen was added to Vero-E6 cells in 12-well plates, and cells were incubated for 7 days before formalin fixation, permeabilization (0.1% Triton-X100), and staining for LASV proteins using an in-house 5 monoclonal antibody mix as the primary antibody (SPR628). Overall, 80 of 128 (63%) RT-qPCR-positive specimens yielded infectious virus. Infectious virus was most frequently isolated from RT-qPCR-positive conjunctival (26 of 30; 87%) and nasal (25 of 29; 86%) specimens (Figure 1(B), Table S3). Infectious virus was quantified by TCID_50_ on Vero-E6 cells. Of samples with quantifiable levels of virus, highest titres were detected in genital specimens (1.58 × 10^2^–4.64 × 10^5^ TCID_50_/mL; *n* = 10 [all females]), followed by nasal (2.70 × 10^1^–2.57 × 10^4^ TCID_50_/mL; *n* = 21), conjunctival (2.17 × 10^1^–4.70 × 10^3^ TCID_50_/mL; *n* = 21), rectal (2.70 × 10^1^–4.64 × 10^2^ TCID_50_/mL; *n* = 3), and oral (2.17 × 10^1^ TCID_50_/mL; *n* = 1) samples (Figure 1(C)).

Here, we demonstrate viral shedding early in infection in the guinea pig model of LF, including at timepoints representing a period of non-specific signs when patients may not yet seek treatment or when LASV may not be suspected (8 and 12 dpi). Beginning 8 dpi, we detected viral RNA and isolated virus from all specimen types evaluated, including conjunctival, oral, nasal, genital, and rectal swabs. Viral RNA and virus isolation were more frequently detected and respective titres were higher in animals infected with the Josiah strain (lethal in guinea pigs); however, infectious virus was also detected in many samples from animals infected with non-lethal Sauerwald strain, indicating that shedding and transmission risk early in infection are likely independent of disease outcome.

In humans, LASV RNA has been detected in lacrimal fluid [5], saliva [5,6], pharyngeal swabs [6], seminal fluid [6–8], vaginal swabs [5,8] and breast milk [8]; both RNA and infectious virus have been detected in urine [6–10]. Our data appear consistent with these reports and build on them by investigating shedding at times points corresponding to early infection, analyzing nasal specimens, and pairing isolation attempts with viral RNA detection. All specimen types we investigated appear to be viable options for diagnostic RT-qPCR. Regarding transmission risk, conjunctival and nasal specimens yielded infectious virus most often. High titres detected in female genital swabs should be noted, along with potential underestimation of infectious virus in oral and rectal specimens due to challenges in virus isolation and titration [11,12].

Most recently, mucosal shedding and viral persistence were investigated in urine, saliva, lacrimal fluid, vaginal fluid, and seminal fluid from a large cohort of human LF survivors at discharge and serially for up to 2 years post discharge [5]. A low percentage of specimens positive in guinea pigs during acute infection were positive for viral RNA in patients at discharge (5, 9, and 21% for saliva, lacrimal, and vaginal fluid, respectively), indicating that the shedding we observed may be limited to pre-clinical and clinical periods. We detected low levels of viral RNA in only a subset of preputial swabs and were not able to isolate the virus from these samples. Thielebein *et al.* detected viral RNA in 80% of seminal fluid specimens at discharge [5]. The discordance in our findings is likely due to the difference in sample type (preputial swab vs. seminal fluid), resulting in underestimation of sexual transmission risk in the males we evaluated.

To our knowledge, shedding and transmission have not been reported in animal models of disease. Recently, oral, rectal and urinary shedding were characterized in *Mastomys natalensis*, the natural host of LASV [13]. RNA was detected in all 3 sample types; however, in contrast to our findings in guinea pigs, virus was only isolated from urine and bladder swabs, supporting different shedding profiles in the natural host compared to laboratory species developing clinical disease.

Overall, our studies indicate a high frequency of mucosal shedding and infectious virus presence early in infection in all sample types we assessed, and provide key data to consider in infection control and diagnostic assay development for LASV and other pathogenic arenaviruses.

## Supplementary Material

Supplemental Material
