# Supplementary material for: Viral RNA and infectious virus in mucosal specimens from guinea pigs modelling early phases of lethal and non-lethal Lassa fever
Source: Emerg Microbes Infect. 2022 May 23;11(1):1390–3. doi: 10.1080/22221751.2022.2071637 (PMC9132395; doi:10.1080/22221751.2022.2071637)
Supplement: Supplemental Material [file TEMI_A_2071637_SM6695.docx]

**Viral RNA and infectious virus in mucosal specimens from guinea pigs modeling early phases of lethal and non-lethal Lassa fever**

Stephen R. Welch, Sarah C. Genzer, JoAnn D. Coleman-McCray, Jessica R. Harmon, Florine E.M. Scholte, Joel M. Montgomery, Christina F. Spiropoulou, Jessica R. Spengler

Viral Special Pathogens Branch, Division of High-Consequence Pathogens and Pathology, National Center for Emerging and Zoonotic Infectious Diseases, Centers for Disease Control and Prevention, Atlanta, GA, United States

**Supplementary materials and methods**

**Biosafety and ethics**

All work with infectious virus or infected animals was conducted in a biosafety level 4 (BSL-4) laboratory at the Centers for Disease Control and Prevention (CDC). Experiments involving

cDNA encoding viral sequences were performed in accordance with approved Institutional Biosafety Committee protocols. All animal experiments were approved by the CDC Institutional Animal Care and Use Committee and performed in an AAALAC-approved facility.

**Quantitative RT-PCR**

RNA samples were obtained using the MagMAX Pathogen RNA/DNA Kit (RNA eluted in 75 µL; Thermo Fisher Scientific). Genomic DNA was removed using BaseLine Zero DNase (Epicentre), and RT-qPCR was performed using SuperScript III Platinum One-Step RT-qPCR Kit (Invitrogen) with strain-specific primers and probe (Sauerwald fwd – ACTTGTGCCACATGCACACT; Sauerwald rev – TGACACAGCTGCATCAAACA; Sauerwald prb – 6FAM|TCACCCCACATTGTGCCCTGA |IABkFQ; Josiah fwd – GTACTCACATGGGATTGATGTCAC; Josiah rev – CTTCCTTGTGATTCAAGGAGTTTC; Josiah prb – 6FAM| TTCGCTACACAACCGGGCTTGACC |IABkFQ; all Integrated DNA Technologies) targeting the N gene of LASV. Standard curves generated by strain-specific in vitro transcripts were used to quantify viral RNA. RNA transcripts were produced using viral RNA as template for cDNA synthesis using SuperScript™ IV First-Strand Synthesis System (Invitrogen), followed by amplification with Q5 DNA polymerase (NEB) and in vitro transcription and DNase treatment with MegaScript T7 kit (Ambion/Thermo Fisher Scientific). Tissue-specific correction for sample preparation was applied using a commercial assay for eukaryotic 18S rRNA Endogenous Control (Thermo Fisher).

**Clinical monitoring of guinea pigs**

Animals’ body temperatures were monitored using implanted microchip transponders (BMDS IPTT-300). Clinical signs were scored based on 14 parameters: 1 point each for quiet, dull, responsive (QDR) disposition, hunched back or ruffled coat, huddling or burrowing; 2 points each for mild or moderate weakness, dehydration (eye recession), ataxia, circling, tremors, head tilt, weight loss of >15%; 3 points each for severe weakness, abnormal breathing, or anemia; 12 points each for paralysis, frank hemorrhage or bleeding, moribund state, or weight loss of > 25%. Humane endpoint criteria are reached when clinical score is ≥ 12.

**Data analysis and graphics**

All graphs were created in GraphPad Prism (v9.3.1). Significance was calculated using a one-sample *t*-test*.* Comparative levels of viral RNA and titers by specimen type were determined based on samples with detectable RNA or enumerable titers; null values were excluded.
